# Crystal structure of bis­[*S*-hexyl 3-(4-methyl­benzyl­idene)di­thio­carbazato-κ^2^ *N* ^3^,*S*]palladium(II)

**DOI:** 10.1107/S2056989015002236

**Published:** 2015-02-11

**Authors:** M. Sabina Begum, M. Belayet Hossain Howlader, M. Chanmiya Sheikh, Ryuta Miyatake, Ennio Zangrando

**Affiliations:** a Department of Chemistry, Rajshahi University, Rajshahi-6205, Bangladesh; b Department of Applied Chemistry, Faculty of Engineering, University of Toyama, 3190 Gofuku, Toyama 930-8555, Japan; c Center for Environmental Conservation and Research Safety, University of Toyama, 3190 Gofuku, Toyama 930-8555, Japan; d Department of Chemical and Pharmaceutical Sciences, via Giorgieri 1, 34127 Trieste, Italy

**Keywords:** crystal structure, palladium(II) complex, *cis*-ligand configuration

## Abstract

The whole mol­ecule of the title complex, [Pd(C_15_H_21_N_2_S_2_)_2_], is generated by twofold rotational symmetry. The palladium(II) atom exhibits a square-planar coordination geometry, and is located on the crystallographic twofold axis that induces a *cis* configuration of the *N*,*S* chelating ligands. In the crystal, mol­ecules stack along the *c*-axis direction and there are no significant inter­molecular inter­actions present. The structure was refined as an inversion twin with a final BASF parameter of 0.34 (2).

## Related literature   

For the crystal structures of the free Schiff base ligand and of its Ni^II^ complex, see: Howlader *et al.* (2015*a* ▸,*b* ▸). For similar bis­(di­thio­carbazato)Pd complexes with a *cis* configuration of the azomethine N and thiol­ate S atoms, see: Ali *et al.* (2002 ▸); Liu *et al.* (2011 ▸); Duan *et al.* (1998 ▸); Tampouris *et al.* (2007 ▸). For complexes with a *trans* configuration, see: Khaledi & Mohd Ali (2011 ▸); Tampouris *et al.* (2007 ▸); Tarafder *et al.* (2010 ▸).
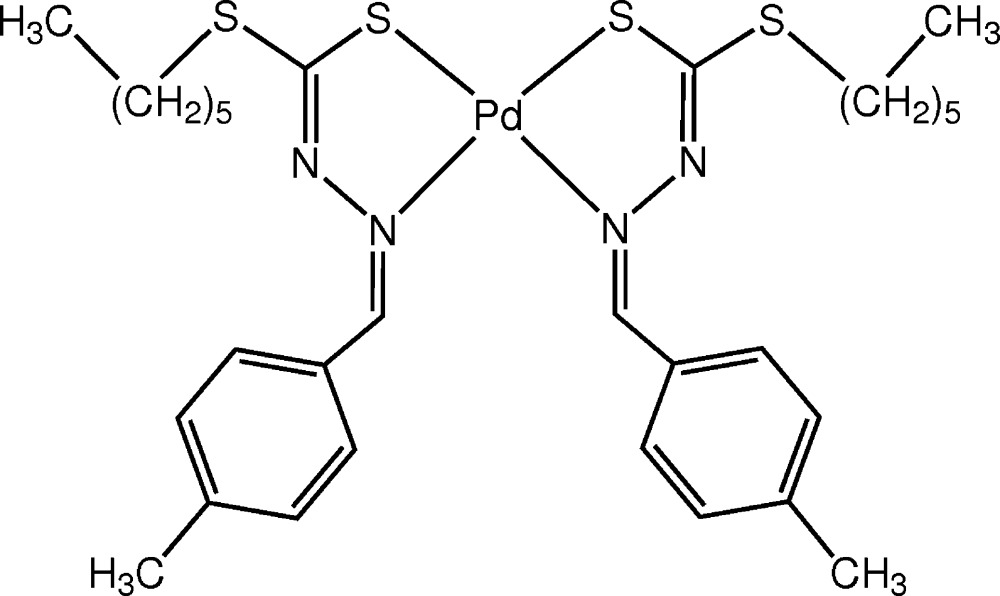


## Experimental   

### Crystal data  

[Pd(C_15_H_21_N_2_S_2_)_2_]
*M* *_r_* = 693.32
Monoclinic, 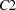

*a* = 18.3559 (11) Å
*b* = 9.6747 (5) Å
*c* = 10.3368 (6) Å
β = 116.810 (2)°
*V* = 1638.37 (16) Å^3^
*Z* = 2
Cu *K*α radiation
μ = 7.14 mm^−1^
*T* = 173 K
0.25 × 0.16 × 0.11 mm

### Data collection  

Rigaku R-AXIS RAPID diffractometer
Absorption correction: multi-scan (*ABSCOR*; Rigaku, 1995 ▸) *T*_min_ = 0.268, *T*_max_ = 0.507
9318 measured reflections
2710 independent reflections
2121 reflections with *I* > 2σ(*I*)
*R*_int_ = 0.120

### Refinement  

*R*[*F* ^2^ > 2σ(*F* ^2^)] = 0.091
*wR*(*F* ^2^) = 0.225
*S* = 1.03
2710 reflections
180 parameters
1 restraint
H-atom parameters constrained
Δρ_max_ = 2.07 e Å^−3^
Δρ_min_ = −1.32 e Å^−3^
Absolute structure: Flack (1983 ▸), 1218 Friedel pairs
Absolute structure parameter: 0.34 (2)

### 

Data collection: *RAPID-AUTO* (Rigaku, 2010 ▸); cell refinement: *RAPID-AUTO*; data reduction: *RAPID-AUTO*; program(s) used to solve structure: *SIR92* (Altomare *et al.*, 1994 ▸); program(s) used to refine structure: *SHELXL97* (Sheldrick, 2015 ▸); molecular graphics: *ORTEP-3 for Windows* (Farrugia, 2012 ▸); software used to prepare material for publication: *PLATON* (Spek, 2009 ▸) and *publCIF* (Westrip, 2010 ▸).

## Supplementary Material

Crystal structure: contains datablock(s) Global, I. DOI: 10.1107/S2056989015002236/su5071sup1.cif

**Fig. 1.**
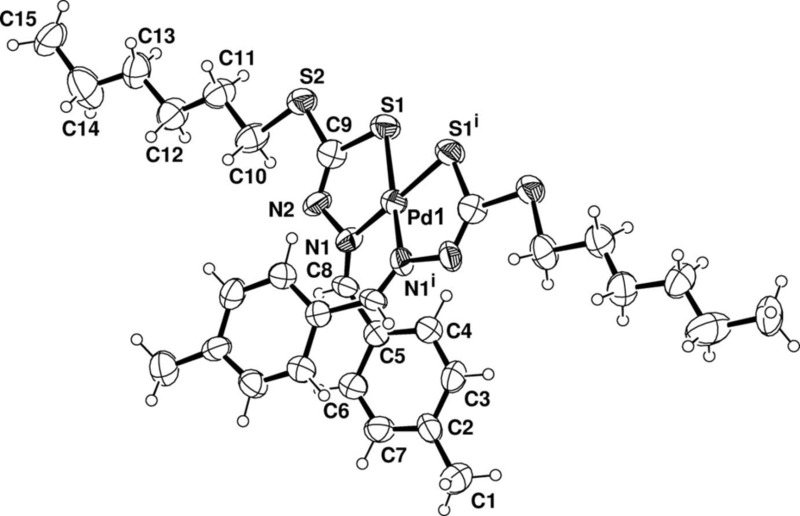
A view of the molecular structure of the title complex, with atom labelling (symmetry code: (i) -x + 1, y, -z). Displacement ellipsoids are drawn at the 50% probability level.

**Fig. 2.**
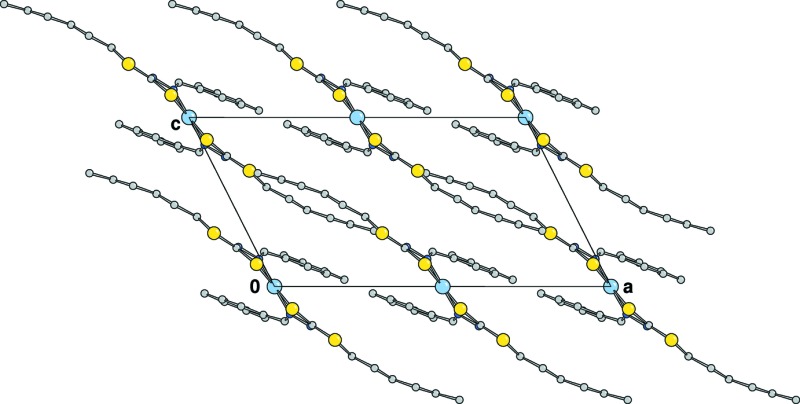
Crystal packing of the title complex viewed along the b axis.

Structure factors: contains datablock(s) I. DOI: 10.1107/S2056989015002236/su5071Isup2.hkl

x y z . DOI: 10.1107/S2056989015002236/su5071fig1.tif
A view of the mol­ecular structure of the title complex, with atom labelling (symmetry code: (i) −*x* + 1, *y*, −*z*). Displacement ellipsoids are drawn at the 50% probability level.

b . DOI: 10.1107/S2056989015002236/su5071fig2.tif
Crystal packing of the title complex viewed along the *b* axis.

CCDC reference: 1046981

Additional supporting information:  crystallographic information; 3D view; checkCIF report

## supplementary crystallographic information

### S1. Synthesis and crystallization

A solution of PdCl_2_ (0.044 g, 0.25 mmol, 25 mL methanol) was added to a solution of the ligand, *S*-hexyl (*E*)-3-(4-methyl­benzyl­idene)di­thio­carbazate (0.147 g, 0.5 mmol, 10 mL methanol). The resulting mixture was stirred at room temperature for 3 h. An orange red precipitate was formed, filtered off, washed with methanol and dried in vacuo over anhydrous CaCl_2_. Orange red single crystals, suitable for X-ray diffraction, of the compound were obtained by slow evaporation from a mixture of chloro­form and aceto­nitrile (1:1) after 5 days (m.p.: 433 K).

### S2. Refinement

Crystal data, data collection and structure refinement details are summarized in Table 1. All H atoms were fixed geometrically (C—H = 0.95 - 0.99 Å) and refined as riding, with *U*_iso_(H) =1.2 *U*_eq_(C). The rather high R factor is affected by the small crystal dimensions and consequently by low diffraction at high θ angles. The collected data were cut at a resolution of 0.85 Å. The structure was refined as an inversion twin with a final BASF parameter = 0.34 (2).

### S3. Comment

In the crystal of the title complex, Fig. 1, the Pd^II^ atom resides on a crystallographic twofold rotational axis and the two chelating Schiff base ligands, in their deprotonated imino thiolate form, coordinate the metal center *via* the azomithine nitrogen atom, N1 and thiolate sulfur atom, S1 in a *cis*-planar configuration (Fig. 1). However, the donor atoms are not coplanar as observed for the corresponding nickel derivative (Howlader *et al.*, 2015*b*), but the square planar geometry presents a slight tetrahedral distortion with displacement of atoms N1 and S1 of ± 0.121 (14) and ∓ 0.134 (6) Å, respectively, from the coordination mean plane. The Pd1–S1 and Pd1—N1 coordination bond distances are of 2.264 (4) and 2.154 (12) Å, respectively, with an N1—Pd1—S1 chelating angle of 83.2 (3)°. These values are in agreement with those observed in similar Pd-bis(dithiocarbazato) complexes, either with a *cis* configuration (Ali *et al.*, 2002; Liu, *et al.*, 2011; Duan *et al.*, 1998; Tampouris *et al.*, 2007) or with a *trans* configuration (Khaledi *et al.*, 2011; Tampouris *et al.*, 2007; Tarafder *et al.*, 2010). It is worth noting that the E conformation about the imine bond N1═C8 [= 1.207 (17) Å; torsion angle N2—N1—C8—C5 = 172.1 (14)°] is different to that observed in the nickel derivative mentioned above, *viz.*1.2 (7)°. This allows an approach between the rings of the methylbenzylidene moieties, with a centroid-to-centroid distance of 4.114 (8) Å, indicating a very weak π-π interaction.

In the crystal, there are no significant intermolecular interactions present. The molecules pack along the *c* axis direction (Fig. 2).

### Crystal data

**Table:**

[Pd(C_15_H_21_N_2_S_2_)_2_]	*Z* = 2
*M**_r_* = 693.32	*F*(000) = 720
Monoclinic, *C*2	*D*_x_ = 1.405 Mg m^−^^3^
Hall symbol: C 2y	Cu *K*α radiation, λ = 1.54187 Å
*a* = 18.3559 (11) Å	θ = 4.8–67.0°
*b* = 9.6747 (5) Å	µ = 7.14 mm^−^^1^
*c* = 10.3368 (6) Å	*T* = 173 K
β = 116.810 (2)°	Prism, orange
*V* = 1638.37 (16) Å^3^	0.25 × 0.16 × 0.11 mm

### Data collection

**Table:**

Rigaku R-AXIS RAPID diffractometer	2710 independent reflections
Radiation source: fine-focus sealed tube	2121 reflections with *I* > 2σ(*I*)
Graphite monochromator	*R*_int_ = 0.120
Detector resolution: 10.000 pixels mm^-1^	θ_max_ = 65.1°, θ_min_ = 4.8°
ω scans	*h* = −21→21
Absorption correction: multi-scan (*ABSCOR*; Rigaku, 1995)	*k* = −11→11
*T*_min_ = 0.268, *T*_max_ = 0.507	*l* = −12→12
9318 measured reflections	

### Refinement

**Table:**

Refinement on *F*^2^	Secondary atom site location: difference Fourier map
Least-squares matrix: full	Hydrogen site location: inferred from neighbouring sites
*R*[*F*^2^ > 2σ(*F*^2^)] = 0.091	H-atom parameters constrained
*wR*(*F*^2^) = 0.225	*w* = 1/[σ^2^(*F*_o_^2^) + (0.1251*P*)^2^] where *P* = (*F*_o_^2^ + 2*F*_c_^2^)/3
*S* = 1.03	(Δ/σ)_max_ < 0.001
2710 reflections	Δρ_max_ = 2.07 e Å^−^^3^
180 parameters	Δρ_min_ = −1.31 e Å^−^^3^
1 restraint	Absolute structure: Flack (1983), 1218 Friedel pairs
Primary atom site location: structure-invariant direct methods	Absolute structure parameter: 0.34 (2)

### Special details

**Table:**

Geometry. All s.u.'s (except the s.u. in the dihedral angle between two l.s. planes) are estimated using the full covariance matrix. The cell s.u.'s are taken into account individually in the estimation of s.u.'s in distances, angles and torsion angles; correlations between s.u.'s in cell parameters are only used when they are defined by crystal symmetry. An approximate (isotropic) treatment of cell s.u.'s is used for estimating s.u.'s involving l.s. planes.
Refinement. Refinement of *F*^2^ against ALL reflections. The weighted *R*-factor *wR* and goodness of fit *S* are based on *F*^2^, conventional *R*-factors *R* are based on *F*, with *F* set to zero for negative *F*^2^. The threshold expression of *F*^2^ > 2σ(*F*^2^) is used only for calculating *R*-factors(gt) *etc*. and is not relevant to the choice of reflections for refinement. *R*-factors based on *F*^2^ are statistically about twice as large as those based on *F*, and *R*- factors based on ALL data will be even larger.

### Fractional atomic coordinates and isotropic or equivalent isotropic displacement parameters (Å^2^)

**Table:**

	*x*	*y*	*z*	*U*_iso_*/*U*_eq_	
Pd1	0.5000	0.5495	0.0000	0.0607 (4)	
S1	0.4807 (3)	0.3802 (4)	0.1325 (5)	0.0725 (11)	
S2	0.4003 (2)	0.3956 (4)	0.3156 (4)	0.0726 (9)	
N1	0.4949 (7)	0.6818 (12)	0.1634 (13)	0.060 (3)	
N2	0.4526 (9)	0.6244 (17)	0.2347 (16)	0.071 (4)	
C1	0.7185 (10)	1.1510 (17)	0.0408 (17)	0.087 (4)	
H1A	0.7670	1.1034	0.0465	0.130*	
H1B	0.7353	1.2327	0.1036	0.130*	
H1C	0.6839	1.1796	−0.0594	0.130*	
C2	0.6712 (6)	1.0545 (19)	0.0900 (11)	0.063 (3)	
C3	0.6745 (8)	0.9122 (16)	0.0758 (15)	0.072 (4)	
H3	0.7083	0.8746	0.0369	0.086*	
C4	0.6290 (7)	0.8236 (13)	0.1178 (14)	0.062 (3)	
H4	0.6339	0.7262	0.1124	0.075*	
C5	0.5759 (7)	0.8797 (13)	0.1679 (13)	0.061 (3)	
C6	0.5741 (8)	1.0255 (14)	0.1841 (14)	0.070 (4)	
H6	0.5412	1.0656	0.2234	0.084*	
C7	0.6196 (8)	1.1052 (14)	0.1431 (14)	0.071 (3)	
H7	0.6161	1.2025	0.1509	0.085*	
C8	0.5215 (8)	0.7962 (12)	0.2045 (14)	0.062 (3)	
H8	0.5048	0.8378	0.2700	0.074*	
C9	0.4449 (11)	0.4926 (16)	0.2274 (18)	0.059 (4)	
C10	0.3742 (9)	0.529 (2)	0.4125 (15)	0.080 (5)	
H10A	0.4242	0.5722	0.4885	0.096*	
H10B	0.3406	0.6025	0.3449	0.096*	
C11	0.3247 (9)	0.4534 (18)	0.4821 (16)	0.081 (4)	
H11A	0.2809	0.3981	0.4062	0.097*	
H11B	0.3617	0.3886	0.5571	0.097*	
C12	0.2885 (8)	0.545 (2)	0.5472 (14)	0.081 (3)	
H12A	0.2580	0.6183	0.4765	0.097*	
H12B	0.3327	0.5904	0.6324	0.097*	
C13	0.2311 (9)	0.4735 (19)	0.5952 (19)	0.091 (5)	
H13A	0.1951	0.4112	0.5161	0.109*	
H13B	0.2642	0.4148	0.6800	0.109*	
C14	0.1813 (12)	0.559 (3)	0.632 (2)	0.133 (7)	
H14A	0.1500	0.6224	0.5504	0.160*	
H14B	0.2165	0.6168	0.7170	0.160*	
C15	0.1205 (10)	0.475 (2)	0.670 (2)	0.110 (6)	
H15A	0.0888	0.5391	0.6982	0.165*	
H15B	0.1512	0.4118	0.7499	0.165*	
H15C	0.0835	0.4224	0.5845	0.165*	

### Atomic displacement parameters (Å^2^)

**Table:**

	*U*^11^	*U*^22^	*U*^33^	*U*^12^	*U*^13^	*U*^23^
Pd1	0.0664 (7)	0.0506 (7)	0.0761 (7)	0.000	0.0419 (5)	0.000
S1	0.093 (3)	0.051 (2)	0.095 (3)	0.001 (2)	0.062 (2)	0.004 (2)
S2	0.084 (2)	0.059 (2)	0.092 (2)	−0.0072 (17)	0.0542 (19)	0.0009 (17)
N1	0.055 (6)	0.057 (7)	0.076 (7)	−0.011 (5)	0.037 (6)	0.006 (5)
N2	0.067 (8)	0.072 (9)	0.091 (9)	−0.027 (7)	0.050 (7)	−0.017 (7)
C1	0.081 (9)	0.094 (12)	0.096 (11)	−0.013 (8)	0.048 (9)	0.004 (8)
C2	0.063 (6)	0.054 (7)	0.073 (6)	0.014 (9)	0.032 (5)	−0.003 (9)
C3	0.063 (8)	0.084 (11)	0.086 (9)	0.003 (7)	0.048 (7)	−0.002 (7)
C4	0.058 (6)	0.048 (7)	0.082 (8)	0.003 (5)	0.034 (6)	0.003 (6)
C5	0.060 (7)	0.052 (7)	0.076 (8)	0.000 (6)	0.036 (6)	0.001 (5)
C6	0.076 (7)	0.053 (10)	0.093 (8)	0.001 (7)	0.048 (7)	−0.005 (6)
C7	0.074 (8)	0.055 (8)	0.080 (9)	−0.004 (6)	0.032 (7)	0.011 (6)
C8	0.079 (8)	0.030 (6)	0.096 (9)	0.006 (6)	0.057 (7)	0.005 (6)
C9	0.068 (9)	0.052 (9)	0.063 (9)	−0.004 (7)	0.034 (7)	0.000 (6)
C10	0.085 (8)	0.090 (14)	0.081 (8)	−0.017 (10)	0.053 (7)	0.007 (9)
C11	0.084 (9)	0.083 (11)	0.086 (10)	−0.009 (8)	0.048 (8)	0.002 (8)
C12	0.084 (7)	0.088 (9)	0.081 (7)	−0.019 (12)	0.047 (6)	−0.019 (12)
C13	0.082 (10)	0.104 (12)	0.109 (12)	0.010 (9)	0.063 (9)	0.002 (9)
C14	0.162 (16)	0.118 (16)	0.150 (15)	0.043 (19)	0.098 (14)	−0.003 (17)
C15	0.106 (12)	0.112 (14)	0.160 (17)	−0.024 (10)	0.102 (13)	−0.010 (11)

### Geometric parameters (Å, º)

**Table:**

Pd1—N1	2.154 (12)	C6—C7	1.337 (16)
Pd1—N1^i^	2.154 (12)	C6—H6	0.9500
Pd1—S1	2.264 (4)	C7—H7	0.9500
Pd1—S1^i^	2.264 (4)	C8—H8	0.9500
S1—C9	1.777 (17)	C10—C11	1.573 (19)
S2—C9	1.747 (16)	C10—H10A	0.9900
S2—C10	1.829 (18)	C10—H10B	0.9900
N1—C8	1.208 (16)	C11—C12	1.44 (2)
N1—N2	1.404 (17)	C11—H11A	0.9900
N2—C9	1.282 (15)	C11—H11B	0.9900
C1—C2	1.511 (19)	C12—C13	1.52 (2)
C1—H1A	0.9800	C12—H12A	0.9900
C1—H1B	0.9800	C12—H12B	0.9900
C1—H1C	0.9800	C13—C14	1.41 (2)
C2—C7	1.381 (16)	C13—H13A	0.9900
C2—C3	1.39 (2)	C13—H13B	0.9900
C3—C4	1.397 (18)	C14—C15	1.56 (3)
C3—H3	0.9500	C14—H14A	0.9900
C4—C5	1.403 (16)	C14—H14B	0.9900
C4—H4	0.9500	C15—H15A	0.9800
C5—C6	1.423 (17)	C15—H15B	0.9800
C5—C8	1.460 (16)	C15—H15C	0.9800
			
N1—Pd1—N1^i^	107.1 (6)	N2—C9—S2	124.7 (15)
N1—Pd1—S1	83.2 (3)	N2—C9—S1	125.6 (15)
N1^i^—Pd1—S1	168.1 (3)	S2—C9—S1	109.6 (9)
N1—Pd1—S1^i^	168.1 (3)	C11—C10—S2	105.5 (13)
N1^i^—Pd1—S1^i^	83.2 (3)	C11—C10—H10A	110.6
S1—Pd1—S1^i^	87.3 (2)	S2—C10—H10A	110.6
C9—S1—Pd1	95.1 (5)	C11—C10—H10B	110.6
C9—S2—C10	101.8 (7)	S2—C10—H10B	110.6
C8—N1—N2	114.2 (12)	H10A—C10—H10B	108.8
C8—N1—Pd1	131.8 (10)	C12—C11—C10	114.0 (15)
N2—N1—Pd1	114.0 (9)	C12—C11—H11A	108.7
C9—N2—N1	115.8 (16)	C10—C11—H11A	108.7
C2—C1—H1A	109.5	C12—C11—H11B	108.7
C2—C1—H1B	109.5	C10—C11—H11B	108.7
H1A—C1—H1B	109.5	H11A—C11—H11B	107.6
C2—C1—H1C	109.5	C11—C12—C13	113.9 (18)
H1A—C1—H1C	109.5	C11—C12—H12A	108.8
H1B—C1—H1C	109.5	C13—C12—H12A	108.8
C7—C2—C3	117.7 (14)	C11—C12—H12B	108.8
C7—C2—C1	121.0 (16)	C13—C12—H12B	108.8
C3—C2—C1	121.2 (12)	H12A—C12—H12B	107.7
C2—C3—C4	120.9 (12)	C14—C13—C12	116.7 (19)
C2—C3—H3	119.5	C14—C13—H13A	108.1
C4—C3—H3	119.5	C12—C13—H13A	108.1
C3—C4—C5	119.4 (12)	C14—C13—H13B	108.1
C3—C4—H4	120.3	C12—C13—H13B	108.1
C5—C4—H4	120.3	H13A—C13—H13B	107.3
C4—C5—C6	118.9 (11)	C13—C14—C15	112 (2)
C4—C5—C8	123.5 (11)	C13—C14—H14A	109.1
C6—C5—C8	117.6 (11)	C15—C14—H14A	109.1
C7—C6—C5	118.9 (12)	C13—C14—H14B	109.1
C7—C6—H6	120.5	C15—C14—H14B	109.1
C5—C6—H6	120.5	H14A—C14—H14B	107.8
C6—C7—C2	124.0 (14)	C14—C15—H15A	109.5
C6—C7—H7	118.0	C14—C15—H15B	109.5
C2—C7—H7	118.0	H15A—C15—H15B	109.5
N1—C8—C5	129.2 (12)	C14—C15—H15C	109.5
N1—C8—H8	115.4	H15A—C15—H15C	109.5
C5—C8—H8	115.4	H15B—C15—H15C	109.5

Symmetry code: (i) −*x*+1, *y*, −*z*.

## References

[bb1] Ali, M. A., Mirza, A. H., Butcher, R. J., Tarafder, M. T. H., Keat, T. B. & Ali, A. M. (2002). *J. Inorg. Biochem.* **92**, 141–148. 10.1016/s0162-0134(02)00559-7 12433421

[bb2] Altomare, A., Cascarano, G., Giacovazzo, C., Guagliardi, A., Burla, M. C., Polidori, G. & Camalli, M. (1994). *J. Appl. Cryst.* **27**, 435.

[bb3] Duan, C.-Y., Tian, Y.-P., Liu, Z.-H., You, X.-Z. & Mak, T. C. W. (1998). *J. Organomet. Chem.* **570**, 155–162.

[bb4] Farrugia, L. J. (2012). *J. Appl. Cryst.* **45**, 849–854.

[bb5] Flack, H. D. (1983). *Acta Cryst.* A**39**, 876–881.

[bb6] Howlader, M. B. H., Begum, M. S., Sheikh, M. C., Miyatake, R. & Zangrando, E. (2015*a*). *Acta Cryst.* E**71**, o103–o104. 10.1107/S2056989015000080 PMC4384598 25878852

[bb7] Howlader, M. B. H., Begum, M. S., Sheikh, M. C., Miyatake, R. & Zangrando, E. (2015*b*). *Acta Cryst.* E**71**, m26–m27. 10.1107/S2056989015000328 PMC4384570 25878838

[bb8] Khaledi, H. & Mohd Ali, H. (2011). *Acta Cryst.* E**67**, m230. 10.1107/S1600536811001991 PMC3051733 21522888

[bb9] Liu, Z.-D., Zhang, X.-J., Wu, J.-Y., Hao, F.-Y., Zhou, H.-P. & Tian, Y.-P. (2011). *Polyhedron*, **30**, 279–283.

[bb10] Rigaku (1995). *ABSCOR*. Rigaku Corporation, Tokyo, Japan.

[bb11] Rigaku (2010). *RAPID AUTO*. Rigaku Corporation, Tokyo, Japan.

[bb12] Sheldrick, G. M. (2015). *Acta Cryst.* C**71**, 3–8.

[bb13] Spek, A. L. (2009). *Acta Cryst.* D**65**, 148–155. 10.1107/S090744490804362X PMC2631630 19171970

[bb14] Tampouris, K., Coco, S., Yannopoulos, A. & Koinis, S. (2007). *Polyhedron*, **26**, 4269–4275.

[bb15] Tarafder, M. T. H., Islam, M. A. A. A. A., Howlader, M. B. H., Guidolin, N. & Zangrando, E. (2010). *Acta Cryst.* C**66**, m363–m365. 10.1107/S0108270110044008 21123878

[bb16] Westrip, S. P. (2010). *J. Appl. Cryst.* **43**, 920–925.

