# Supplementary figures and images for: Crystal structure of bis­[S-hexyl 3-(4-methyl­benzyl­idene)di­thio­carbazato-κ2 N 3,S]palladium(II)
Source: Acta Crystallogr E Crystallogr Commun. 2015 Feb 11;71(Pt 3):m63–4. doi: 10.1107/S2056989015002236 (PMC4350759; doi:10.1107/S2056989015002236)

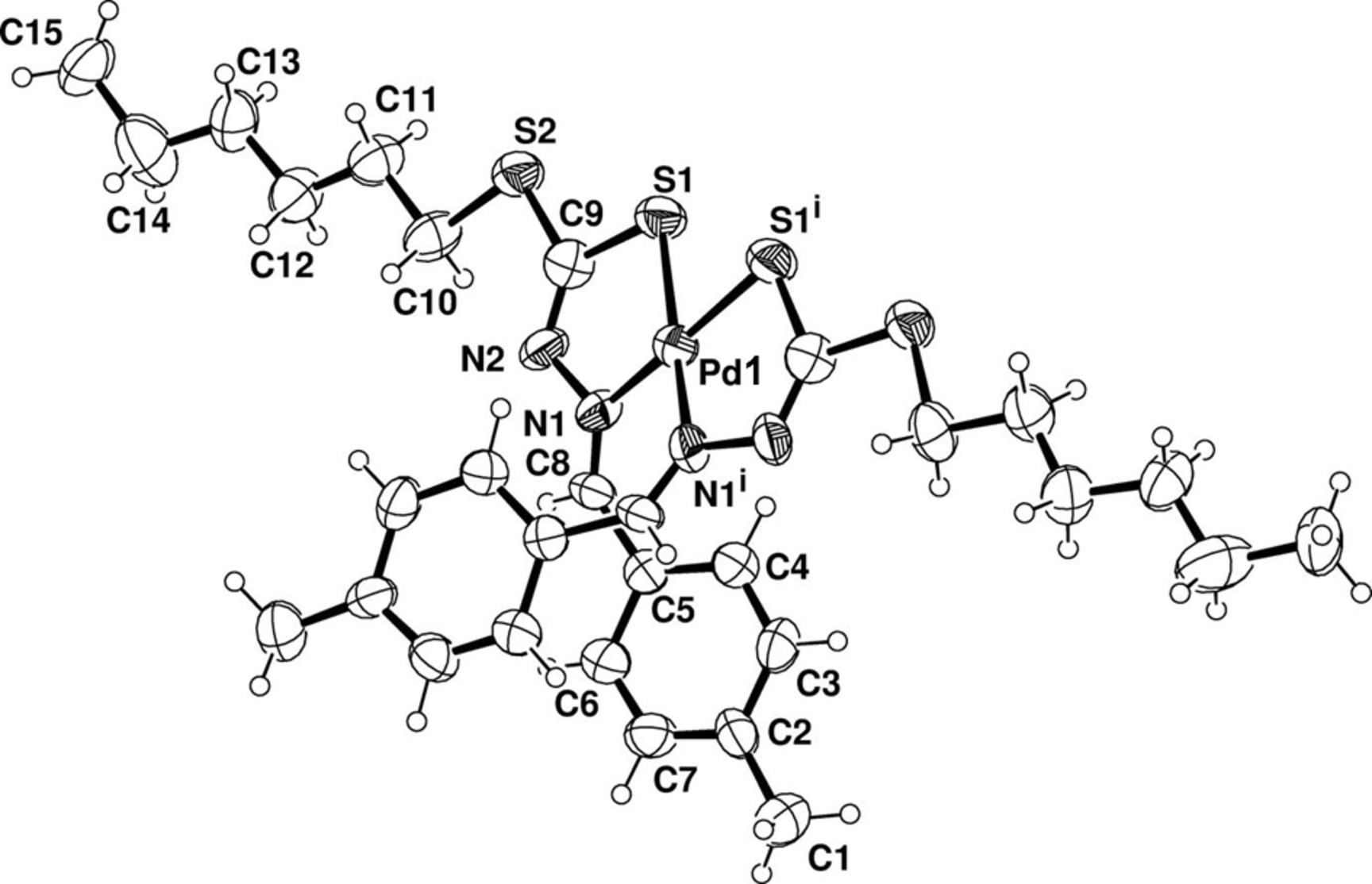

Supplement: Supplementary file 3 [file e-71-00m63-fig1.tif]

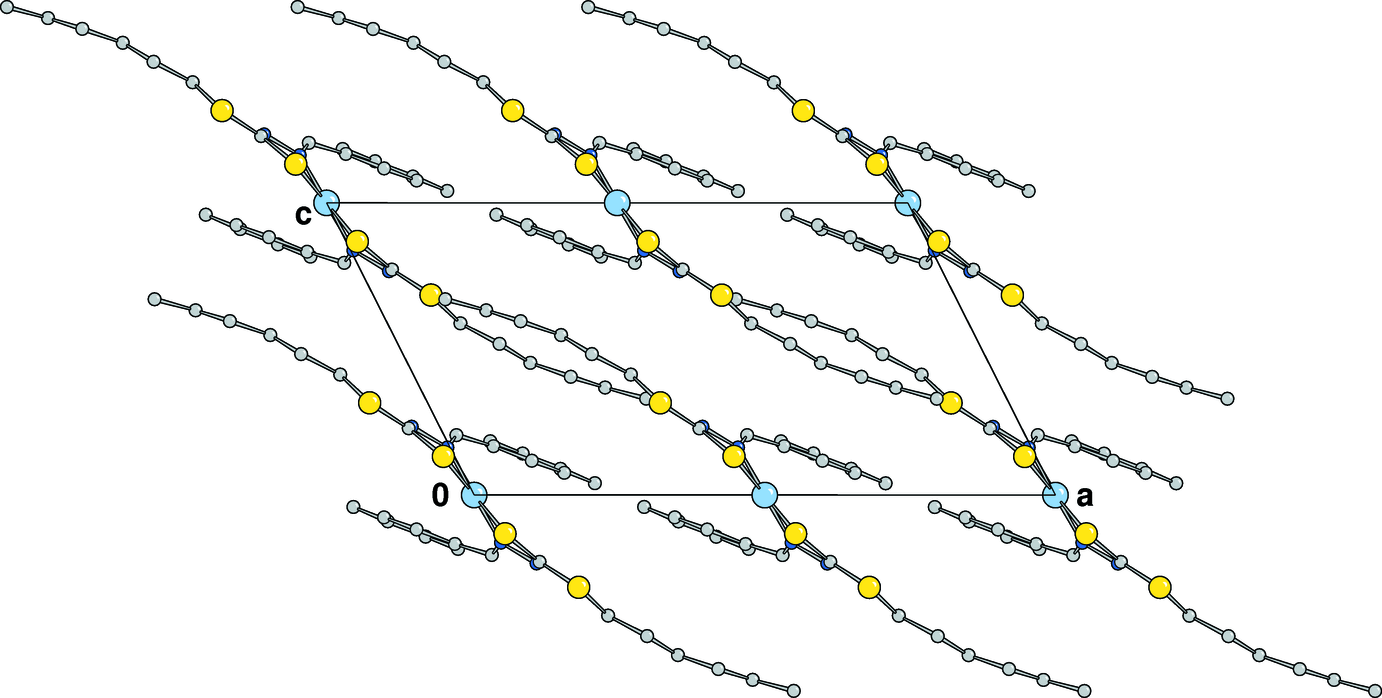

Supplement: Supplementary file 4 [file e-71-00m63-fig2.tif]
